# Correction: Hydroxy-directed iridium-catalyzed enantioselective formal β-C(sp^2^)–H allylic alkylation of α,β-unsaturated carbonyls

**DOI:** 10.1039/d5sc90128f

**Published:** 2025-06-11

**Authors:** Sankash Mitra, Rahul Sarkar, Aditya Chakrabarty, Santanu Mukherjee

**Affiliations:** a Department of Organic Chemistry, Indian Institute of Science Bangalore 560 012 India sm@iisc.ac.in +91-80-2360-0529 +91-80-2293-2850

## Abstract

Correction for ‘Hydroxy-directed iridium-catalyzed enantioselective formal β-C(sp^2^)–H allylic alkylation of α,β-unsaturated carbonyls’ by Sankash Mitra *et al.*, *Chem. Sci.*, 2022, **13**, 12491–12497, https://doi.org/10.1039/D2SC03966D.

The authors regret that in the original article, the absolute configuration of 3-phenyldihydrofuranone 8 was assigned to be (*R*) by comparing its specific rotation with that reported in the literature.^1^ Accordingly, the absolute configuration of 3aa was assigned as (*R*) and those of the other products (3), shown in Tables 2 and 3, were inferred as the same by analogy.

However, recent literature reports^2,3^ revealed the absolute configuration of 8 to be (*S*), as shown below:
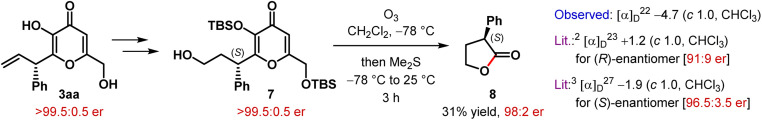


Accordingly, the absolute configuration of 3aa was reassigned as (*S*). As a consequence, the newly generated allylic stereocenter in all the β-allyl 3-hydroxypyranone derivatives (shown in Tables 1–3) as well as the compounds derived from them (Scheme 2) in the original article should be opposite.

The corrected structures and data for all the compounds have also been included in the revised ESI. The ESI has been updated as of 02/06/2025 to reflect all these changes.

These corrections, however, do not alter the conclusions of the original article.

The graphical abstract image has also been updated to the following:
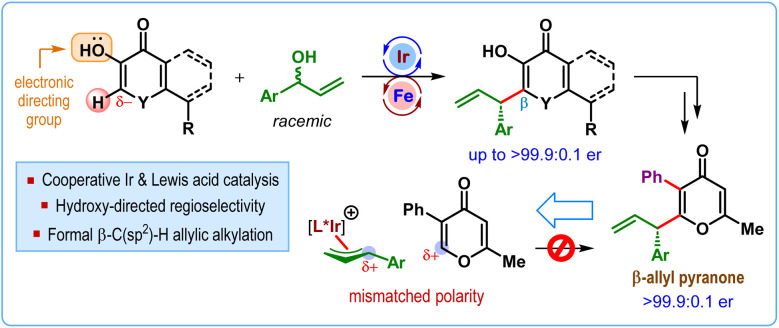


The Royal Society of Chemistry apologises for these errors and any consequent inconvenience to authors and readers.
